# Hypertension in adolescents: The role of obesity and family history

**DOI:** 10.1111/jch.14381

**Published:** 2021-11-16

**Authors:** Weiying Zhao, Luxia Mo, Yusheng Pang

**Affiliations:** ^1^ Department of Pediatrics The First Affiliated Hospital of Guangxi Medical University Nanning Guangxi China

**Keywords:** adolescent, family history, obesity, pediatric hypertension

## Abstract

We evaluated the combined effect of obesity and family history (FH) on the risk of hypertension in adolescents. We studied 1288 school‐aged adolescents aged 16.0 ± 0.5 years (49.0% males) attending the medical examination for enrollment in the city of Nanning, China. Their blood pressure, weight, and height were measured. A questionnaire was administered to both adolescents and their parents to obtain information on the participants’ medical history. Multiple logistic regression analysis, according to bodyweight categories and adjusted for age, gender, and body mass index (BMI), was done to determine the association of FH with hypertension. Hypertension was found in 14.1% of adolescents. The prevalence of hypertension was significantly higher in adolescents with obesity and positive FH than their normal weight and negative FH counterparts. For adolescents with normal weight and waist circumstance (WC), those with a positive FH in parents compared to those without had an significantly increased risk for hypertension (odds ratio [OR], 2.15; 95% confidence interval [CI] 1.28–3.61, and 1.96; 95% CI 1.16–3.32, respectively). These findings were adjusted for age, gender, and BMI. Our study showed that routine screening for pediatric hypertension should be performed in adolescents who are overweight and obese. Furthermore, parental FH of hypertension played an important role in predicting the hypertension phenotype among adolescents with normal weight.

## 1 INTRODUCTION

Hypertension is one of the 10 most common chronic diseases in pediatrics, with increasing rates in recent decades.^1^, ^2^, ^3^ Target organ damage, including left ventricular hypertrophy and carotid intimal medial thickness, has been reported in hypertensive children at initial diagnosis.^4^ Growing evidence indicates that elevated blood pressure (BP) in childhood or adolescence is associated with intermediate phenotypes and adverse cardiovascular outcomes in adults.^5^, ^6^ Hence, timely recognition, diagnosis, and management of pediatric hypertension is necessary to reduce cardiovascular morbidity and mortality.

Elevated BP is categorized into two: primary (essential) and secondary hypertension. In children, hypertension is often attributed to secondary causes, such as renal parenchymal, renovascular, and endocrine etiologies.^7^ Significantly elevated BP and its definitive etiologies contributed to the leading causes of hospitalization in the pediatric population according to our previous studies.^8^ However, essential hypertension remains to be the most common type found among adolescents.

Essential hypertension often presents with modest BP elevations. It is associated with being overweight or obese, a contributory family history (FH), higher dietary sodium intake, and premature birth.^9^ As the earliest manifestation of adult cardiovascular disease, essential hypertension in children has yet to be given more consideration.

A simple and accurate screening method to identify pediatric hypertension is urgently needed. Obesity and positive FH are the major determinants of essential hypertension in adults and children.^10^ Overall and abdominal obesity, measured by body mass index (BMI) and waist circumference (WC), are common alternative measures of adiposity in clinical and public health practice.

Hypertension exhibits familial clustering,^11^, ^12^ and is associated with higher BP in offspring of hypertensive parents or grandparents.^12^, ^13^ However, there is a paucity of data in the joint effect of FH and obesity on the risk of hypertension. In this study, we assessed the combined effect of obesity and a positive FH in estimating the risk of hypertension in healthy Chinese adolescents.

## 2 METHODS

### 2.1 Study design and population

The participants included 1316 high school freshmen. The study was based on cross‐sectional baseline data of their medical examinations for enrollment to a large‐scale boarding school conducted on September 2020 in Nanning, Guangxi, Southwest China. The study aimed to provide insight on the impact of genetic and environmental factors influencing complex traits, such as obesity, hypertension, and dyslipidemia during adolescence. Individuals were excluded if there were (1) signs and symptoms of infectious or chronic diseases, which was recognized through the assessment of clinical presentation, accurate physical examination, and medical history, (2) any prior history of medication use within 2 weeks, which might influence the measurements of BP levels; and (3) insufficient data on FH and anthropometric measurement in their medical reports.

The project was conducted in accordance with the Declaration of Helsinki guidelines and approved by the clinical ethical committee of the First Affiliated Hospital of Guangxi Medical University. Written informed consent was obtained from the parents prior to the enrollment of individuals in the study.

### 2.2 Anthropometrics and BP measurements

The anthropometric measurements were performed according to standard protocols. Bodyweight and height were measured with light clothing and without shoes. WC was measured at the midpoint between the right lower rib and the iliac crest, and after a slight breath out. Weight was measured in kilograms (kg) and rounded off to 2 decimal places. Height and WC were measured to the nearest 0.1 centimeter (cm). BMI was calculated as weight in kg/ (height in meters)^2^. Systolic and diastolic BP (SBP and DBP, respectively) were measured three times over a 5‐minute period using a digital automatic monitor (OMRON, HEM‐7124) on the right arm after 15 minutes of seated rest. The appropriate BP cuff size was used for each individual. The average of the last two BP readings for each individual was used in the final analysis.

### 2.3 Definition of obesity and hypertension

Overweight and obesity were defined as BMI above the 85th and 95th percentiles, respectively, of age‐ and sex‐specific reference values for Chinese adolescents.^14^

Abdominal obesity was defined as a WC above the 85th percentiles of age‐ and sex‐specific reference values.^15^ Elevated BP and hypertension were defined as either an SBP or DBP above the 90th and the 95th percentiles, respectively, of age‐, sex‐, and height‐specific reference values for Chinese adolescents.^16^

### 2.4 Family history (FH) of essential hypertension (EH) definition

A “complete” FH consists of three generations, including the study participants, their parents as first‐degree relatives, and grandparents as second‐degree relatives. A positive FH of EH was defined as at least one biological parent or grandparent with EH. A negative FH was defined as the individuals’ parents and all the grandparents were negative for EH. Diagnosis of EH (ie, BP of 140/90 mmHg, on anti‐hypertensive medications, or verified by the individual's physician) was reported by individuals and their parents through questionnaires, further confirmed through remote consultation. If a parent or grandparent had passed away, diagnosis of EH was determined by whether he had hypertension before his death.

### 2.5 Statistical analyses

General statistical analyses were carried out using Statistical Package for the Social Science (SPSS) software version 22.0 (SPSS, Chicago, Illinois, USA). Descriptive characteristics are presented as mean ± standard deviation (SD), median ± interquartile range (IQR), or percentage (%). Chi‐square test was used for qualitative data. Continuous data were tested for normality using the Kolmogorov‐Smirnov test, and Equality of variances was assessed using Levene's test. To increase the statistical power, elevated BP and hypertension were together used as dependent variables.

Multiple logistic regression analysis was performed to determine the association of FH with high BP, according to bodyweight categories. The analysis was adjusted for age, sex, and BMI. We assessed our models using residual normal quantile plots and examined for differences in the beta estimate and differences in fits. Moreover, the individuals were divided into groups according to a positive or negative FH, and a nonparametric rank‐based statistical test, such as the Mann–Whitney *U* test for comparisons between groups and Kruskal‐Wallis test for multi‐class data, was performed. Results with *P*‐values < .05 were considered statistically significant.

## 3 RESULTS

### 3.1 Descriptive statistics for the participants

Table 1 presents the prevalence of hypertension in our study participants based on different characteristics. Our sample was comprised 1288 adolescents with a mean age of 16.5±0.5 years (49.0% males) that had complete data for anthropometric indices, BP measurements, and FH according to our study protocol. Prevalence of hypertension was significantly higher in adolescents with overweight and obesity, than their normal‐weight counterparts (18.2% vs 31.4% vs 11.9%, *P* < .001).

**TABLE 1:** The prevalence of hypertension of study populations

Variables	Individuals	Hypertension (%)	χ^2^	*P*
Total	1288	181 (14.1)		
Age, years	16.5 ± 0.5	–		
Gender			17.829	<.001
Male	631	115 (18.2)		
Female	657	66 (10.0)		
BMI status			28.131	<.001
Normal weight	1021	121 (11.9)		
Overweight	181	33 (18.2)		
Obesity	86	27 (31.4)		
Abdominal obesity			12.740	<.001
No	994	121 (12.2)		
Yes	294	60 (20.4)		
FH			6.215	.013
No	925	116 (12.5)		
Yes	363	65 (17.9)		
Members of positive FH				
0	925	116 (12.5)	10.823	.013
1	251	43 (17.1)		
2	83	13 (15.7)		
≥3	29	9 (31.0)		

*Note*: Data are presented as mean ± SD for age, counts (percentages) for categorical variables.

Abbreviations: BMI, body mass index; FH, Family history.

**FIGURE 1:**
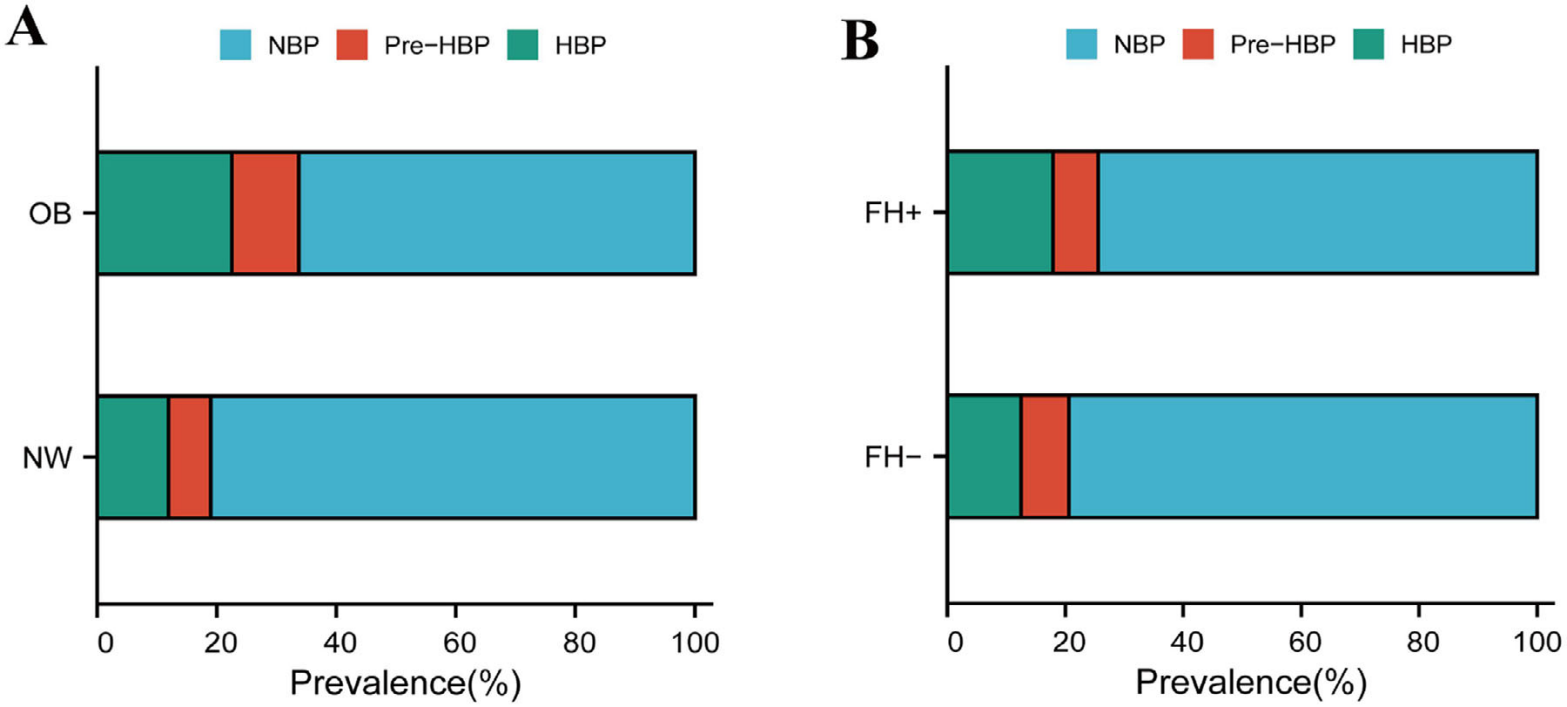
Distribution of blood pressure status according to obesity and FH status. (A) The prevalence of NBP, Pre‐HBP, and HBP stratified by two weight categories (66.3%, 11.2%, 22.5% vs 81.0%, 7.1%, 11.9%, *P* < .001); (B) the prevalence of NBP, Pre‐HBP, and HBP stratified by two categories of FH ((74.4%, 7.7%, 17.9% vs 79.4%, 8.1%, 12.5%, *P *= .045). Abbreviations: OB, overweight /obesity; NW, underweight/normal weight; FH+, Positive FH of EH; FH‐, Negative FH of EH; NBP, normal blood pressure; Pre‐HBP, elevated BP; HBP, hypertension

Prevalence of hypertension was also significantly higher in those with positive FH of hypertension than those without FH(17.9% vs 12.5%, *P *= .013). Those with a higher number of family members with EH had greater hypertension prevalence (12.5% vs 17.1% vs 15.7% vs 31.0%, *P *= .013). Figure 1 shows the prevalence of normal BP, elevated BP, and hypertension stratified by body weight categories and FH status.

### 3.2 Association of FH with hypertension in offspring

Logistic regression analysis results shown in Table 2 revealed that a positive FH is significantly associated with hypertension among the total population, particularly among the adolescents with normal weight. Compared to negative FH counterparts with normal weight, an increased risk for hypertension was observed in the offspring with positive FH in parents (odds ratio [OR], 2.15; 95% confidence interval [CI] 1.28–3.61), after controlling for age, gender, and BMI. However, for adolescents with overweight and obesity, positive FH of hypertension was not significantly associated with elevated BP. Similar patterns were observed in abdominal obesity (OR, 1.96; 95% CI 1.16–3.32).

**TABLE 2:** Logistic regression association of family history with hypertension in adolescents stratified by obesity status

		FH, only in grandparents		FH, in parents	
Variables	Negative FH	OR (95%CI), *P*	OR*(95%CI), P*	OR (95%CI), *P*	OR*(95%CI), P*
Normal weight	Reference	1.20(0.81,1.79), .360	1.22(0.81,1.82), .341	2.12(1.28,3.53), .004	2.15(1.28,3.61), .004
Overweight and Obesity	Reference	0.91(0.45,1.84), .795	0.95(0.46,1.98), .896	1.05(0.51,2.16), .892	1.28(0.59,2.76), .529
Total	Reference	1.10(0.78,1.56), .571	1.15(0.81,1.63), .442	1.83(1.21,2.76), .004	1.76(1.14,2.71), .010
Normal waist circumference	Reference	1.33(0.65,1.43), .839	1.29(0.87,1.92), .203	1.95(1.17,3.27), .011	1.96(1.16,3.32), .012
Abdominal obesity	Reference	0.65(0.30,1.39), .263	1.36(0.67,2.75), .393	0.70(0.32,1.56), .385	1.44(0.68,3.04), .342
Total	Reference	1.10(0.78,1.56), .571	1.15(0.81,1.63), .442	1.83(1.21,2.76), .004	1.76(1.14,2.71), .010

*Note*: OR, Odds ratio; CI, confidence interval; *P*, unadjusted; P*, adjusted for age, gender, and BMI.

Abbreviations: FH, family history of hypertension. FH, only in grandparents: at least one grandparent diagnosed with hypertension without such a parental history; FH, in parents: at least one parent diagnosed with hypertension irrespective of FH status of grandparents.

### 3.3 Comparison of participants with different FH status

Analyses of the anthropometrics of individuals with different FH status is shown in Table 3. The individuals with positive FH show a higher BMI, WC, SBP, and DBP (*P *= .092, *P *= .438, *P *= .055, and *P *= .003, respectively). Furthermore, individuals with positive FH involving grandparents showed a higher trend on parameters compared to those with negative FH (although not statistically significant). In addition, offspring with positive FH involving parents, showed the highest WC, BMI, and DBP, irrespective of FH status of grandparents (*P *= .003, *P *= .001, and *P *= .011, respectively).

**TABLE 3:** The demographic and anthropometric measures of offspring stratified by FH group status

Parameters	All (No. = 1288)	Normotension in parents and grandparents (No. = 925)	Hypertensive parents or grandparents (No. = 363)	*P*	No hypertension in parents and grandparents (No. = 925)	FH, only in grandparents (No. = 242)	FH, in parents (No. = 121)	*P*
Age, years^a^	16.5 ± 0.5	16.5 ± 0.5	16.5 ± 0.5	.129	16.5 ± 0.5	16.5 ± 0.5	16.6 ± 0.6	.232
Gender (F/M)	657/631	461/464	196/167	.216	461/464	129/113	67/54	.237
SBP, mmHg^b^	112 ± 12	111 ± 16	113 ± 15	.055	111 ± 16	113 ± 14	112 ± 17	.155
DBP, mmHg^b^	70 ± 8	69 ± 9	71 ± 11	.003	69 ± 9	71 ± 11	70 ± 10*	.011
BMI, kg/m^2^ ^b^	20.52 ± 3.55	20.44 ± 4.11	20.77 ± 4.09	.092	20.44 ± 4.10	20.55 ± 3.51	21.19 ± 5.10*, **	.001
WC, cm^b^	69.7 ± 9.9	68.5 ± 10.6	70.0 ± 10.9	.438	69.5 ± 10.5	69.5 ± 10.5	72.5 ± 12.3*, **	.003

Abbreviations: BMI, body mass index; FH, in parents: at least one parent diagnosed with hypertension irrespective of FH status of grandparents.; FH, only in grandparents: at least one grandparent diagnosed with hypertension without such a parental history; SBP, systolic blood pressure, DBP, diastolic blood pressure; WC, waist circumference.

Note: ^a^Data was presented as mean ± SD for normal distribution.

^b^Data was presented as median ± IQR for skewed distribution.

* *P* < .05 vs No hypertension in parents and grandparents.

** *P* < .05 vs FH, only in grandparents; *P* < .05, between‐group differences.

## 4 DISCUSSION

In the present study, we investigated the combined effect of obesity and FH status in estimating the risk of hypertension in Chinese adolescents. Adolescents with BMI classified as either overweight or obese were associated with a higher prevalence of hypertension than their normal‐weight counterparts. Moreover, individuals with positive FH had an increaed risk of hypertension than those without. Meanwhile, individuals with positive FH involving their parents exhibited the highest values of WC, BMI, and DBP. Logistic regression analysis further revealed that positive FH involving parents was significantly associated with hypertension in the total population, particularly for adolescents with normal weight.

Although not statistically significant, individuals of positive FH showed higher BMI, WC, SBP, and DBP. These findings are in agreement with Roberto and associates,^17^ who found that an offspring of at least one hypertensive parent showed higher BMI, SBP, and DBP. A Chinese study including 6049 children and adolescents aged 6–17 years also showed that those with a history of hypertension in one parent or both parents were more likely to develop hypertension (OR, 1.28; CI 1.01‐1.61, and OR, 2.24; CI 1.09‐4.61, respectively), adjusted for sex, age, BMI, parents’ age, and educational qualifications, compared with children and adolescents whose parents had no history of hypertension.^18^ However, other studies have reported the opposite.^19^, ^20^ A school‐based study from Turkey including 2166 students aged 6–15 years found that a positive FH of hypertension was not associated with hypertension in children.^19^ Another Chinese case‐control study including 3266 students aged 11–16 years also found no association between FH in parents and hypertension in univariate and obesity‐stratified analyses.^20^ The discrepancies of these findings may be caused by methodological differences, including the variations in statistical methods and the criteria used for high BP. More recently, studies further reported that children with a FH of hypertension had significantly higher insulin levels, lipids, and greater risk of being overweight,^21^, ^22^, ^23^ particularly those with a positive maternal FH.^22^, ^24^ This supports the hypothesis that a cardiovascular risk phenotype is transmitted on the maternal lineage.

Hypertension is a chronic progressive disease, demonstrating higher prevalence rates with increasing age. In China, the prevalence of hypertension ranges from 5% to 30% from childhood to adulthood.^3^, ^25^ Adolescence, with a high prevalence of multiple cardiovascular risk factors,^26^ is an important phase. The individuals in our study were adolescents aged 11–18 years, while the mean age of their parents was less than 50 years. We also evaluated whether a positive FH involving only grandparents was associated with increased BP in adolescents. In our univariate analysis, positive FH in grandparents showed a slightly mild effect on parameters compared with individuals of negative FH. This is consistent with a case‐control observational pedigree study of 153 families in the United States,^12^ which found that there was no significant difference in the OR between the FH beyond parents and familial EH in children. In contrast to our study's finding, data from a multi‐generational cohort of the Framingham Heart Study showed that early‐onset hypertension in grandparents increased the risk of developing hypertension in their grandchildren by 33%.^13^ The results were significant after controlling for early‐onset hypertension in parents and other lifestyle factors. However, grandparents with late‐onset hypertension were not associated with an increased risk for hypertension in their grandchildren. The authors defined early‐onset hypertension as being diagnosed at an age less than 55 years. This may account for the inconsistencies noted between our studies.

Compared with previous studies, the prevalence of hypertension among overweight and obese individuals was similar to that of Maldonado and associates who reported that hypertension was greater in obese individuals (23%), than in overweight (14%) and normal weight (8%) healthy Portuguese children and adolescents. In this study, FH is significantly related to an increased risk of hypertension in the total sample and normal‐weight adolescents. Having a positive FH may account for a significant fraction of disease burden in adolescents who are within normal‐weight conditions. Further, obesity status accounts for a larger fraction of the disease burden among obese adolescents.^27^ Together, our studies suggest that genetic factors may play a role in inter‐individual susceptibility to high BP prior to the onset of obesity. FH captures the overt results of shared genetics (common and rare genetic variants) and shared environmental risks (ie, dietary intake) among family members. Therefore, with this modulatory effect of obesity in mind, patients with elevated BP and normal weight may become the novel targets of future studies to explore the genetic variants of hypertension.

The study has some limitations. First, FH data were collected from self‐reported questionnaires and were not objectively measured. Second, confounding factors, such as diet, physical inactivity, and socioeconomic status, that are well‐established risk factors for hypertension, were not adjusted for in our study. Third, BP measurements were based on a single visit, which may be responsible for the higher prevalence of high BP observed in our study. Ideally, elevated BP must be confirmed on repeated measures before a child is diagnosed with hypertension.

## 5 CONCLUSION

Early routine screening for pediatric hypertension should be performed in adolescents with a BMI classification of overweight or obesity. Furthermore, adolescents with a normal BMI and a positive FH involving their parents should be paid for ample attention in routine prevalence screening. Our findings provided evidence that FH of EH in parents played a significant role in predicting the hypertension phenotype among adolescents with normal weight.

## CONFLICTS OF INTERESTS

The authors have no conflicts of interest to disclose.

## AUTHOR CONTRIBUTIONS

Zhao W. and Pang Y. contributed to the research conception and design. Zhao W. and Mo L. contributed to data acquisition, analysis, and interpretation. Zhao W. and Pang Y. drafted and revised the article. All authors had final approval of the submitted and published versions.
